# Social Media, Public Health, and Community Mitigation of COVID-19: Challenges, Risks, and Benefits

**DOI:** 10.2196/36804

**Published:** 2022-04-12

**Authors:** Corey H Basch, Charles E Basch, Grace C Hillyer, Zoe C Meleo-Erwin

**Affiliations:** 1 Department of Public Health William Paterson University Wayne, NJ United States; 2 Department of Health and Behavior Studies Teachers College Columbia University New York, NY United States; 3 Department of Epidemiology Mailman School of Public Health Columbia University New York, NY United States; 4 see Acknowledgments

**Keywords:** COVID-19 pandemic, social media, misinformation, disinformation, COVID-19, pandemic, infodemiology, health literacy, health information, public health, COVID risk, information seeking

## Abstract

Shortly after the first case reports in 2019, COVID-19 was declared a pandemic. Early messages from trusted experts, which later proved to be inadequate or incorrect, highlight the need for continual adjustment of messages to the public as scientific knowledge evolves. During this time, social media exploded with greatly sought-after information, some of which was misinformation based on incomplete or incorrect facts or disinformation purposefully spread to advance a specific agenda. Because of the nature of social media, information, whether accurate or not at the time posted, lives on and remains accessible to the public even when its usefulness has been discredited. While the impact of mis/disinformation on COVID-19 risk-reducing behaviors is debatable, it is clear that social media has played a significant role in both extending the reach of COVID-19–related falsehoods and promoting evidence-based content. Over the last decade, social media has become a dominant source of information that consumers turn to for health information. A great deal of misinformation and disinformation has reached large numbers of social media users, which points to a need for the agencies of the US Public Health Service to create communications to convey accurate and current information and appeals that will actually be viewed. This viewpoint highlights the challenges, risks, and potential benefits that social media present in mitigating the COVID-19 pandemic.

## Background

At the close of 2019, SARS-CoV-2, the virus that causes COVID-19, emerged in Wuhan, China, and rapidly spread throughout the world, being labeled a pandemic in only a few months after its emergence [1,2]. Nearly 2 years after the initial outbreak, as of April 8, 2022, there have been 496,355,574 confirmed cases of COVID-19, 6,170,720 cumulative deaths, and 11,085,254,518 vaccine doses administered globally [3]. The Omicron variant is also circulating globally, quickly driving up cases in regions that had previously seen reprieve owing in part to high rates of vaccination [4,5].

The only way to prevent COVID-19 from occurring is through both reducing exposure to SARS-CoV-2 and reducing susceptibility. Since COVID-19 is primarily transmitted through air droplets and aerosols [6,7], the main strategies for reducing exposure are social distancing [8], mask use [9,10], and limiting time spent in poorly ventilated spaces where air is shared with other people [11,12]. The main strategy for reducing susceptibility is vaccination [13,14]. With respect to COVID-19 prevention, individuals’ personal decision-making about behaviors to reduce their susceptibility and/or exposure influences risk at both the individual and community-wide levels [15,16].

Individuals’ health and illness–related decision-making is affected by many different sources of influence such as family members and friends, health care providers, political leaders, and the media. A plethora of behavioral science theories demonstrate how individuals’ levels of knowledge and beliefs, skills, availability and accessibility to resources, and social support shape health choices [17-21]. Over the past decade, examination of the role played by social media in personal decisions that affect individual, family, and population health, including those related to infectious disease, has expanded rapidly. This viewpoint highlights the challenges, risks, and potential benefits that social media present in mitigating the COVID-19 pandemic.

## Challenges

Airborne transmission of droplets and aerosols is the main way SARS-CoV-2 is transmitted [6,7]. Secondarily, although far less frequent, the virus can also be transmitted through droplets and aerosols settling on fomites (inanimate objects) and having hand/nose or hand/mouth contact [22]. In the early stage of the pandemic, emphasis was placed on transmission through fomites, and on handwashing and surface disinfection, as well as social distancing. Yet, little to no emphasis was placed on masking. Messaging about fomite transmission was eventually dialed back [23,24], while messaging on mask recommendations was altered entirely.

In times of global and national crisis, the public clamors for the guidance of trusted experts to navigate rapidly evolving and potentially life-threatening situations. Early on, the experts at Centers for Disease Control and Prevention (CDC), the World Health Organization (WHO), and the US Surgeon General blasted messages that subsequently required modification. False messages of conspiracy rapidly surfaced, and charlatans promoted unsubstantiated claims to prevent disease. To some degree, future expert messages were discredited.

For example, on February 29, 2020, US Surgeon General Dr Jerome Adams used Twitter to send the following message to the public: “Seriously people- STOP BUYING MASKS! They are NOT effective in preventing general public from catching #Coronavirus, but if healthcare providers can’t get them to care for sick patients, it puts them and our communities at risk!” [25]. This sentiment was in concert with the early mentality of the CDC, WHO, US Office of the Surgeon General, and other constituents at the time.

All of these entities would later reverse their views as further scientific evidence emerged regarding the ambient transmission of SARS CoV-2 [26-30]. With spread of the Delta variant during the summer of 2021, CDC messaging encouraged unvaccinated individuals to wear masks in crowded public places and for vaccinated individuals to wear masks indoors in areas of high transmission [31]. When the Omicron variant began to spread in late 2021, news reports began appearing with quotes from health experts advising the public to upgrade their masks [32-34]. However, the official CDC guidance on masking was not clarified until mid-January 2022 [31].

In a context of rapidly evolving scientific discovery, a key challenge for public health education is to adapt messaging in an information environment containing older content that continues to live and circulate online despite being obsolete or inaccurate from the outset.

A second major challenge is that public health experts may (and often do) disagree about recommendations. Communicating the nuances of scientific progress may be especially hampered by the fact that nearly 60% of the population has medium or low scientific literacy [35]. Given the uncertainty involved in an emerging global pandemic, the incremental and often contentious way in which scientific knowledge typically advances, and shifting and diverging recommendations, it is not surprising that public confusion is common and trust in official governmental agencies is hindered [35].

## Risks

Large numbers of individuals search for health information online [36,37]. As with previous infectious diseases [38], the public turned to the internet for information on the novel coronavirus [39-41]. Unfortunately, false information about the COVID-19 pandemic on social media has been extensive [42-44] and viewed by millions of people [45]. False information involves both misinformation, or inaccurate conclusions about a phenomenon drawn from incomplete or incorrect facts, as well as disinformation, which pertains to the purposeful spread of false information in line with a specific agenda [46]. The early days of the pandemic saw such a proliferation of false information online, leading the WHO Director General to declare that the fight was not only against the pandemic but against an “infodemic” as well [47]. An “infodemic” can be described as “an overabundance of information – some accurate and some not – that makes it hard for people to find trustworthy sources and reliable guidance when they need it” [47].

Preventing COVID-19 involves both reducing exposure to SARS-CoV-2 through masking, social distancing, and the avoidance of crowded, poorly ventilated spaces, as well as through reducing susceptibility via vaccination. In the United States, there are two types of vaccines available: mRNA vaccines and an adenovirus-based vaccine [48,49]. It has become clear that one of the concerns that the public has about the vaccine pertains to the comparatively short time spent on development, testing, and approval (emergency use authorization). Officials sought to assure the public that neither safety nor scientific integrity was compromised in the process [50-52]. Nevertheless, public concern that mRNA would alter one’s genes and fear surrounding an untested technology persisted [53,54]. Surveys in the United States about attitudes toward vaccination (whether for oneself or one’s children) continue to reveal persistent levels of hesitancy and refusal [55], with notable demographic and political differences [56-60].

The trustworthiness of online information about COVID-19 vaccination is endangered by those whose aim is to spread disinformation. Disinformation can be spread by anyone. One estimate was that 12 individuals were responsible for 65% of the mis/disinformation spread on Twitter, Instagram, and Facebook [61]. These individuals were identified as “anti-vaccine activists, alternative health entrepreneurs and physicians” [61] who spread a wealth of antivaccination content over the course of the pandemic [62]. The danger of mis/disinformation is epitomized by its use “to incite violence and crime targeted at ethnic minorities – which has resulted in deaths and displacement of children, led to lower child COVID vaccination rates, undermined trust in journalism and science, and drowned out marginalized voices” [63]. Mis/disinformation in times of health emergencies threatens national security and there have been calls for immediate intervention [64]; however, this is not easily accomplished in a societal context with freedom of speech and access to digital communication channels capable of reaching millions of people in minutes.

A small number of social media accounts spreading disinformation have had widespread reach and have gained many followers during the pandemic [62]. To the extent that this small number of antivaccination messengers attract many users who are skeptical or are “on the fence” about vaccination, they can undermine efforts to mitigate community transmission and increase the risk of more pathogenic and virulent variants.

The influence of misinformation and disinformation on acceptance of inaccurate COVID-19 information, behavioral intentions, and actual behavior remain equivocal [65-67]. Some evidence suggests that such communications are associated with acceptance of COVID-19 conspiracy theories [65,68] and other inaccurate information [69]. A belief in conspiracy theories may reduce the uptake of at least some preventative behaviors [70,71].

In sum, while the behavioral impact of misinformation and disinformation on social media is debated, what is clear is the role that social media has played in extending the reach of COVID-19–related falsehoods. Of course, even if low-quality or blatantly untrue COVID-19 online information has a minimal impact on COVID-19 exposure and susceptibility reduction, in the case of a highly contagious respiratory virus, the impact on community spread may nevertheless be of considerable concern.

## Benefits of a Multifaceted Public Health Presence on Social Media

It has been well-established that social media can spread accurate, useful information. During the pandemic, social media companies have claimed to make efforts to remove harmful COVID-19 content and promote accurate information [72-74], although the degree to which they have been successful in doing so has been questioned [75]. Previous investigations have demonstrated examples of how social media have promoted positive behaviors such as the Global Handwashing Partnership [76]. Some research suggests that although misleading COVID-19 information has been widely shared on social media, evidence-based content may have been shared to a greater extent. Fortunately, public health agencies such as the WHO and CDC have increased their presence on key social media platforms [77]. Beyond creating their own content, public health agencies have begun to look to social media personalities to help disseminate prevention messages. For example, in 2020, the state of Texas moved to pay influencers to promote mask use and social distancing on TikTok [78].

We previously identified a shift in popular YouTube videos regarding the source of messages on COVID-19 from news media [79] to entertainers [80]. From January to March 2020, there was a large increase in the number and proportion of cumulative views garnered by widely viewed videos on hand hygiene, from 33,268,243 (26.6%) to 182,331,135 (51.3%) [80], and there was an increase coverage of face masks in the most popular videos [80]. Our work examining mechanisms of disease transmission across three successive samples indicated an increase from ~63 million views to more than 273 million views for videos specifically mentioning disease transmission. This increase coincided with a rise in the worldwide number of cases and the occurrence of COVID-19 transmission [81]. A Medline search performed at the time of writing this viewpoint indicated that these are among the first successive samples of YouTube videos published to date. Our studies suggest that tracking changes over time is useful and that social media may help to amplify scientific recommendations. Notably, successive sampling captures evolving situations rather than labeling something as misinformation simply because it was posted at a time when science about the topic was evolving.

Similar to YouTube, there is active conversation about COVID-19 on TikTok. Earlier research suggested that, despite 1 billion views with the #Coronavirus hashtag, there was minimal discussion about transmission [82]. However, with more directed searching, we identified messaging about prevention [83]. Notably, a search of the hashtag #WearAMask indicated that the number of views of TikTok videos mentioning masks uploaded by the WHO was far less than those in the “Wear a Mask” campaign, despite the fact that the messages from the WHO were more aligned with scientific recommendations [6].

Humor, music, and dance were large components of the #WearAMask posts, but were not common in those from the WHO [83]. Consistent with the study on YouTube, these findings suggest that popular entertainers and the use of nonconventional approaches to messaging can help draw attention to public health messages. While conversations on TikTok could be health-promoting (eg, using masks, social distancing, testing, handwashing) [83-85], they may also be counterproductive as was noted in our study of misinformation related to vaccination [86]. Thus, while there are challenges with and cause for concern regarding COVID-19 content on social media, social media clearly play a vital role in disseminating accurate information.

## Moving Forward

Public health communication best practices suggest that in cases where science is not definitive, messages should be calibrated to make it clear that there is an evolving context [87]. Our work indicates that the content of official public health agency social media accounts does not receive as many views as communications posted by popular entertainers, influencers, or, in some cases, even consumers. Partnerships with carefully vetted content creators may help to extend the reach of accurate health information on social media, particularly to young people who tend to use these platforms in higher numbers than older adults [88].

Because there is a propensity for research highlighting misinformation as a means to gain attention [89,90], the dialog should be clear about what constitutes misinformation. Researchers should consider the date that material was posted and how scientific understanding changed over time, rather than aim to produce “clickbait” titles. There are degrees of misinformation and calibrating messages with a disclaimer will also help in this circumstance. Studies claiming they have identified misinformation should be forthcoming about how this was coded and what factors were taken into consideration, with concrete examples of what did and did not constitute misinformation and disinformation.

Effective communication and education should be geared toward a very heterogenous public in terms of age, beliefs, culture, sociodemographics, literacy, and information-seeking sources. Over the last decade, social media have become a dominant source of information that consumers turn to for health and COVID-19 information. This situation prompted our early work to determine what is and is not being communicated. The findings to date suggest there is considerable room for improvement. A great deal of misinformation and disinformation has reached large numbers of social media users, pointing to a need for the agencies of the US Public Health Service to create communications to convey accurate and current information and appeals that will actually be viewed.

## Abbreviations

CDC: Centers for Disease Control and Prevention
WHO: World Health Organization

## References

[1] Wu Y, Chen C, Chan Y (2020). The outbreak of COVID-19: an overview. J Chin Med Assoc.

[2] (2020). WHO Director-General's opening remarks at the media briefing on COVID-19. World Health Organization.

[3] Coronavirus disease (COVID-19) pandemic. World Health Organization.

[4] Araf Y, Akter F, Tang Y, Fatemi R, Parvez MSA, Zheng C, Hossain MG (2022). Omicron variant of SARS-CoV-2: Genomics, transmissibility, and responses to current COVID-19 vaccines. J Med Virol.

[5] Smith Rogers L (2022). John Hopkins Bloomberg School of Mental Health.

[6] (2021). Framework for implementation of COVID-19 community mitigation measures for lower-resource countries. Centers for Disease Control and Prevention.

[7] Tang S, Mao Y, Jones RM, Tan Q, Ji JS, Li N, Shen J, Lv Y, Pan L, Ding P, Wang X, Wang Y, MacIntyre CR, Shi X (2020). Aerosol transmission of SARS-CoV-2: evidence, prevention and control. Environ Int.

[8] Morawska L, Milton DK (2020). It is time to address airborne transmission of Coronavirus Disease 2019 (COVID-19). Clin Infect Dis.

[9] (2021). Centers for Disease Control and Prevention.

[10] Van Dyke ME, Rogers TM, Pevzner E, Satterwhite CL, Shah HB, Beckman WJ, Ahmed F, Hunt DC, Rule J (2020). Trends in county-level COVID-19 incidence in counties with and without a mask mandate - Kansas, June 1-August 23, 2020. MMWR Morb Mortal Wkly Rep.

[11] (2021). Scientific Brief: Community use of cloth masks to control the spread of SARS-CoV-2. Centers for Disease Control and Prevention.

[12] (2021). Ventilation in buildings. Centers for Disease Control and Prevention.

[13] Morawska L, Tang JW, Bahnfleth W, Bluyssen PM, Boerstra A, Buonanno G, Cao J, Dancer S, Floto A, Franchimon F, Haworth C, Hogeling J, Isaxon C, Jimenez JL, Kurnitski J, Li Y, Loomans M, Marks G, Marr LC, Mazzarella L, Melikov AK, Miller S, Milton DK, Nazaroff W, Nielsen PV, Noakes C, Peccia J, Querol X, Sekhar C, Seppänen O, Tanabe S, Tellier R, Tham KW, Wargocki P, Wierzbicka A, Yao M (2020). How can airborne transmission of COVID-19 indoors be minimised?. Environ Int.

[14] Hodgson SH, Mansatta K, Mallett G, Harris V, Emary KRW, Pollard AJ (2021). What defines an efficacious COVID-19 vaccine? A review of the challenges assessing the clinical efficacy of vaccines against SARS-CoV-2. Lancet Infect Dis.

[15] (2021). Centers for Disease Control and Prevention.

[16] Fischhoff B (2020). Making decisions in a COVID-19 world. JAMA.

[17] Norheim O, Abi-Rached J, Bright L, Bærøe K, Ferraz OLM, Gloppen S, Voorhoeve A (2021). Difficult trade-offs in response to COVID-19: the case for open and inclusive decision making. Nat Med.

[18] Green L, Kreuter M (2005). Health program planning: An educational and ecological approach. 4th edition.

[19] Prochaska J, Redding C, Evers K, Glanz K, Rimer BK, Viswanath K (2002). The transtheoretical model stages of change. Health behavior: theory, research, and practice. 3rd edition.

[20] Rosenstock IM (1974). Historical origins of the health belief model. Health Educ Monograph.

[21] Rogers E (2003). Diffusion of innovations, 5th edition.

[22] Glanz K, Bishop DB (2010). The role of behavioral science theory in development and implementation of public health interventions. Annu Rev Public Health.

[23] (2021). How COVID-19 spreads. Centers for Disease Control and Prevention.

[24] Goldman E (2020). Exaggerated risk of transmission of COVID-19 by fomites. Lancet Infect Dis.

[25] (2020). CDC updates COVID-19 transmission webpage to clarify information about types of spread. Centers for Disease Control and Prevention.

[26] Jingnan H (2020). Why there are so many different guidelines for face masks for the public. NPR Goats and Soda.

[27] Yu IT, Li Y, Wong TW, Tam W, Chan AT, Lee JH, Leung DY, Ho T (2004). Evidence of airborne transmission of the severe acute respiratory syndrome virus. N Engl J Med.

[28] Friedman U (2020). Face masks are in. The Atlantic.

[29] Felter C, Bussemaker N (2020). Which countries are requiring face masks?. Council on Foreign Relations.

[30] Manjoo F (2020). How the world's richest country ran out of a 75-cent face mask. The New York Times.

[31] (2022). Types of masks and respirators. Centers for Disease Control and Prevention.

[32] Khaled F (2021). Should Americans 'upgrade' face coverings to surgical masks amid Omicron spread?. Newsweek.

[33] Caron C (2021). Is It safe to travel? Can I still see my family on Christmas?. New York Times.

[34] Godoy M, Doucleff M, Wroth C (2021). Don't let omicron crash your holiday gathering. Here's how to keep your family safe. NPR.

[35] Kennedy B, Hefferon M (2019). What Americans know about science. Pew Research Center.

[36] Fox S (2014). The social life of health information. Pew Research Center.

[37] (2013). The internet and health. Pew Research Center.

[38] Fidler DP (2019). Disinformation and disease: social media and the Ebola epidemic in the Democratic Republic of the Congo. Council on Foreign Relations.

[39] Timoneda J, Vallejo Vera S (2021). Will I die of coronavirus? Google Trends data reveal that politics determine virus fears. PLoS One.

[40] Cinarka H, Uysal M, Cifter A, Niksarlioglu EY, Çarkoğlu A (2021). The relationship between Google search interest for pulmonary symptoms and COVID-19 cases using dynamic conditional correlation analysis. Sci Rep.

[41] O'Leary D, Storey V (2020). A Google–Wikipedia–Twitter model as a leading indicator of the numbers of coronavirus deaths. Intell Sys Acc Fin Mgmt.

[42] Romer D, Jamieson KH (2020). Conspiracy theories as barriers to controlling the spread of COVID-19 in the U.S. Soc Sci Med.

[43] (2020). Carnegie Mellon University.

[44] Hamel L, Lopes L, Kirzinger A, Sparks G, Stokes M, Brodie M (2021). KFF COVID-19 Vaccine Monitor: media and misinformation. Kaiser Family Foundation.

[45] Obiała J, Obiała K, Mańczak M, Owoc J, Olszewski R (2021). COVID-19 misinformation: accuracy of articles about coronavirus prevention mostly shared on social media. Health Policy Technol.

[46] Igoe KJ Establishing the truth: vaccines, social media, and the spread of misinformation. Harvard TH Chan School of Public Health.

[47] Understanding the infodemic and misinformation in the fight against COVID-19. Pan American Health Organization.

[48] (2022). Different COVID-19 vaccines. Centers for Disease Control and Prevention.

[49] (2021). FDA issues emergency use authorization for third COVID-19 vaccine. US Food and Drug Administration.

[50] Maragakis L, Kelen GB (2022). Is the COVID-19 vaccine safe?. Johns Hopkins Medicine.

[51] Owens C (2021). Why the coronavirus vaccine rollout is behind schedule. Axios.

[52] (2020). Historical vaccine safety concerns. Centers for Disease Control and Prevention.

[53] Chiu A, Bever L (2021). Are they experimental? Can they alter DNA? Experts tackle lingering coronavirus vaccine fears. The Washington Post.

[54] Cohen J (2021). Further evidence supports controversial claim that SARS-CoV-2 genes can integrate with human DNA. Science.

[55] Hamel L, Lopes L, Sparks G, Kirzinger A, Kearney A, Stokes M, Brodie M (2021). KFF COVID-19 Vaccine Monitor: October 2021. Kaiser Family Foundation.

[56] Kates J, Tolbert J, Orgera K (2021). The red/blue divide in COVID-19 vaccination rates. Kaiser Family Foundation.

[57] Beleche T, Ruhter J, Kolbe A, Marus J, Bush L, Sommers B (2021). COVID-19 vaccine hesitancy: demographic factors, geographic patterns, and changes over time. Assistant Secretary for Planning and Evaluation (ASPE).

[58] Ndugga N, Hill L, Artiga S, Haldar S (2022). Latest data on COVID-19 vaccinations by race/ethnicity. Kaiser Family Foundation.

[59] Kirzinger A, Sparks G, Kearney A, Stokes M, Hamel L, Brodie M (2021). KFF COVID-19 Vaccine Monitor: November 2021. Kaiser Family Foundation.

[60] KFF COVID-19 Vaccine Monitor: Does the public want to get a COVID-19 vaccine? When?. Kaiser Family Foundation.

[61] Bond S (2021). Just 12 people are behind most vaccine hoaxes on social media, research shows. NPR.

[62] (2021). The disinformation dozen. Center for Countering Digital Hate.

[63] Vosloo S (2021). Digital misinformation/disinformation and children. UNICEF.

[64] Meeting COVID-19 misinformation/disinformation head-on. Johns Hopkins Bloomberg School of Public Health.

[65] Romer D, Jamieson KH (2021). Patterns of media use, strength of belief in COVID-19 conspiracy theories, and the prevention of COVID-19 from March to July 2020 in the United States: survey study. J Med Internet Res.

[66] Hornik R, Kikut A, Jesch E, Woko C, Siegel L, Kim K (2021). Association of COVID-19 misinformation with face mask wearing and social distancing in a nationally representative US sample. Health Commun.

[67] Resnicow K, Bacon E, Yang P, Hawley S, Van Horn ML, An L (2021). Novel predictors of COVID-19 protective behaviors among US adults: cross-sectional survey. J Med Internet Res.

[68] De Coninck D, Frissen T, Matthijs K, d'Haenens L, Lits G, Champagne-Poirier O, Carignan M, David MD, Pignard-Cheynel N, Salerno S, Généreux M (2021). Beliefs in conspiracy theories and misinformation about COVID-19: comparative perspectives on the role of anxiety, depression and exposure to and trust in information sources. Front Psychol.

[69] Bridgman A, Merkley E, Loewen P, Owen T, Ruths D, Teichmann L, Zhilin O (2020). The causes and consequences of COVID-19 misperceptions: Understanding the role of news and social media. Harvard Kennedy School Misinformation Review.

[70] van Prooijen J, Etienne T, Kutiyski Y, Krouwel A (2021). Conspiracy beliefs prospectively predict health behavior and well-being during a pandemic. Psychol Med.

[71] Chan H, Chiu CP, Zuo S, Wang X, Liu L, Hong Y (2021). Not-so-straightforward links between believing in COVID-19-related conspiracy theories and engaging in disease-preventive behaviours. Humanit Soc Sci Commun.

[72] Mosseri A (2021). Working to stop misinformation and false news. Meta.

[73] (2021). YouTube to remove all anti-vaccine misinformation. BBC News.

[74] Hernandez J (2022). Facebook suspends Marjorie Taylor Greene's account over COVID misinformation. NPR.

[75] Cadier A (2021). Misinformation 'superspreaders': Covid vaccine falsehoods still thriving on Facebook and Instagram. NewsGuard.

[76] (2021). Social media. Global Handwashing Partnership.

[77] Brown A (2020). Coronavirus: The World Health Organization is becoming the planet's most important social media influencer. Forbes.

[78] Marks M (2020). How state officials are trying to use TikTok to stop the spread Of COVID-19. Texas Standard.

[79] Basch CH, Hillyer GC, Meleo-Erwin ZC, Jaime C, Mohlman J, Basch CE (2020). Preventive behaviors conveyed on YouTube to mitigate transmission of COVID-19: cross-sectional study. JMIR Public Health Surveill.

[80] Basch CE, Basch CH, Hillyer GC, Jaime C (2020). The role of YouTube and the entertainment industry in saving lives by educating and mobilizing the public to adopt behaviors for community mitigation of COVID-19: successive sampling design study. JMIR Public Health Surveill.

[81] Hillyer GC, Basch CH, Basch CE (2021). Coverage of transmission of COVID-19 information on successive samples of YouTube videos. J Community Health.

[82] Basch C, Hillyer G, Jaime C COVID-19 on TikTok: harnessing an emerging social media platform to convey important public health messages. Int J Adolesc Med Health..

[83] Basch CH, Fera J, Pierce I, Basch CE (2021). Promoting mask use on TikTok: descriptive, cross-sectional study. JMIR Public Health Surveill.

[84] Basch CH, Mohlman J, Fera J, Tang H, Pellicane A, Basch CE (2021). Community mitigation of COVID-19 and portrayal of testing on TikTok: descriptive study. JMIR Public Health Surveill.

[85] Basch CH, Fera J, Pellicane A, Basch CE (2022). Handwashing videos on TikTok during the COVID-19 pandemic: potential for disease prevention and health promotion. Infect Dis Health.

[86] Basch CH, Meleo-Erwin Z, Fera J, Jaime C, Basch CE (2021). A global pandemic in the time of viral memes: COVID-19 vaccine misinformation and disinformation on TikTok. Hum Vaccin Immunother.

[87] Jacobsen KH, Vraga EK (2020). Improving communication about COVID-19 and emerging infectious diseases. Eur J Clin Invest.

[88] Auxier B, Anderson M (2021). Social media use in 2021. Pew Research Center.

[89] Lima D, Lopes M, Brito A (2020). Social media: friend or foe in the COVID-19 pandemic?. Clinics (Sao Paulo).

[90] Atehortua NA, Patino S (2021). COVID-19, a tale of two pandemics: novel coronavirus and fake news messaging. Health Promot Int.

